# VPS45 is required for both diffuse and tip growth of *Arabidopsis thaliana* cells

**DOI:** 10.3389/fpls.2023.1120307

**Published:** 2023-02-27

**Authors:** Yosia Mugume, Rahul Roy, William Agbemafle, Gabriella N. Shepard, Yee Vue, Diane C. Bassham

**Affiliations:** ^1^ Department of Genetics, Development and Cell Biology, Iowa State University, Ames, IA, United States; ^2^ Roy J. Carver Department of Biochemistry, Biophysics and Molecular Biology, Iowa State University, Ames, IA, United States

**Keywords:** arabidopsis, endomembrane, tip growth, root hairs, SM protein, vacuole

## Abstract

**Introduction:**

VPS45 belongs to the Sec1/Munc18 family of proteins, which interact with and regulate Qa-SNARE function during membrane fusion. We have shown previously that *Arabidopsis thaliana* VPS45 interacts with the SYP61/SYP41/VTI12 SNARE complex, which locates on the *trans*-Golgi network (TGN). It is required for SYP41 stability, and it functions in cargo trafficking to the vacuole and in cell expansion. It is also required for correct auxin distribution during gravitropism and lateral root growth.

**Results:**

As *vps45* knockout mutation is lethal in Arabidopsis, we identified a mutant, *vps45-3*, with a point mutation in the *VPS45* gene causing a serine 284-to-phenylalanine substitution. The VPS45-3 protein is stable and maintains interaction with SYP61 and SYP41. However, *vps45-3* plants display severe growth defects with significantly reduced organ and cell size, similar to *vps45* RNAi transgenic lines that have reduced VPS45 protein levels. Root hair and pollen tube elongation, both processes of tip growth, are highly compromised in *vps45-3*. Mutant root hairs are shorter and thicker than those of wild-type plants, and are wavy. These root hairs have vacuolar defects, containing many small vacuoles, compared with WT root hairs with a single large vacuole occupying much of the cell volume. Pollen tubes were also significantly shorter in *vps45-3* compared to WT.

**Discussion:**

We thus show that VPS45 is essential for proper tip growth and propose that the observed vacuolar defects lead to loss of the turgor pressure needed for tip growth.

## 1 Introduction

The endomembrane system consists of membrane bound organelles that exchange protein and lipid cargo by vesicle trafficking (Wang and Hussey, 2015; Rout and Field, 2017). Vesicle trafficking proteins mediate vesicle budding from the donor compartment, movement, and docking and fusion with a target organelle (Bonifacino and Glick, 2004). These proteins include SNAREs (soluble *N*-ethylmaleimide sensitive factor adaptor protein receptors), Rab GTPases, tethers, and regulatory Sec1/Munc18 (SM) proteins (Kim and Brandizzi, 2012; Hong and Lev, 2014; Zhang and Hughson, 2021), which cooperate to drive membrane fusion. The cargo contained within a vesicle is therefore delivered to a cellular compartment such as the Golgi or, in the case of exocytosis, released from the cell (Baker and Hughson, 2016).

SNARE proteins catalyze vesicle fusion in all eukaryotes, with formation of a *trans*-SNARE complex between SNAREs on opposing membranes. The complex involves three target SNAREs (t-SNAREs) anchored on the target membrane and a vesicle SNARE (v-SNARE), anchored on the destination membrane (Jahn and Scheller, 2006; Baker and Hughson, 2016). SNAREs are also classified as Qa, Qb, Qc (usually t-SNAREs) and R (usually v-SNARES), depending on the presence of a conserved central glutamine or arginine residue in the SNARE motif (Ungar and Hughson, 2003; Zhang and Hughson, 2021). SNARE-mediated membrane fusion is regulated by SM-family proteins, which are peripheral membrane proteins that interact with Qa-SNAREs (Carr and Rizo, 2010; Rizo and Südhof, 2012). The SM protein VPS (Vacuolar Protein Sorting) 45 localizes to the *trans*-Golgi network and early endosomes and functions in vesicle fusion with these organelles (Koumandou et al., 2007).

In *Arabidopsis thaliana*, homozygous null mutations in *VPS45* are lethal, showing that VPS45 is critical for plant growth. VPS45 interacts with the SYP (Syntaxin of Plants) 41/SYP61/VTI (Vps ten interacting) 12 SNARE complex at the TGN (Zouhar et al., 2009), which regulates trafficking at the TGN for vacuolar cargo sorting, secretion of cell wall components, auxin homeostasis and abiotic/biotic stress responses (Bassham et al., 2000; Zhu et al., 2002; Surpin et al., 2003; Uemura et al., 2012; Tanaka et al., 2013). RNA interference lines with greatly reduced VPS45 protein levels also had decreased SYP41 protein (Zouhar et al., 2009), reminiscent of the situation in yeast, in which Tlg2p, a putative ortholog of SYP41, is unstable in a *vps45* mutant (Bryant and James, 2001). This further underscores the role of VPS45 in regulating the activity of the SYP41/SYP61/VTI12 SNARE complex. VPS45 silencing resulted in mis-sorting of vacuolar sorting receptors (VSRs), membrane-bound receptors that recognize cargo for transport to vacuoles, and interfered with the sorting of cargo containing C-terminal vacuolar sorting determinants (ctVSDs) (Zouhar et al., 2009). *Atben2*, containing a point mutation in *VPS45* resulting in an aspartate-to-asparagine substitution at the 129^th^ amino acid position, revealed a role for VPS45 in auxin transporter recycling and endocytic uptake of membrane cargo from the plasma membrane (Tanaka et al., 2013). Together, these results suggest a critical role for VPS45, along with the SYP41/SYP61/VTI12 complex, at the TGN in endocytic and vacuolar cargo sorting.

Here, we identified *vps45-3*, a *VPS45* mutant harboring a serine to phenylalanine substitution at the 284^th^ position of the polypeptide. *vps45-3* plants have a dwarf phenotype, with reduced organ size and cell expansion defects, consistent with the previously reported RNAi lines (Zouhar et al., 2009). The mutants also have reduced growth of root hairs and pollen tubes, potentially caused by fragmented vacuoles. VPS45 is therefore important for cell expansion in both diffusely growing and tip growing cells.

## 2 Materials and methods

### 2.1 *Arabidopsis thaliana* genotypes and plant growth conditions

*Arabidopsis thaliana* genotypes used are WT (Columbia-0), *vps45-3* (TILLING mutant), *vps45-3* COM (complemented line), WT-EYFP-RabF2a, *vps45-3*-EYFP-RabF2a. All genotypes were grown at 22°C either on soil in growth chambers or on sterile nutrient media under light racks. Soil-grown plants were kept in long day (16 hr light/8 hr dark) conditions. For growth on nutrient media, seeds were surface sterilized in 33% (v/v) bleach and 0.1% (v/v) Triton X-100 (Thermo Scientific, AAA16046AP) solution for 10 minutes and washed with sterile water at least five times. After two days of cold treatment in the dark, the seeds were plated on solid ^1^/_2_-strength Murashige-Skoog (MS) medium with vitamins (Caisson Labs, MSP09), 1% (w/v) sucrose (IBI scientific, IB37160), 2.4 mM 2-morphinolino-ethanesulfonic acid pH 5.7 (Sigma-Aldrich, M3671) and 0.8% (w/v) Phytoagar (Caisson Labs, PTP01). *vps45-3* was generated by TILLING (Colbert et al., 2001) and mutants identified using forward primer 5’ -TGGCGTTGAAACGAAGACCTGTCA-3’ and reverse primer 5’-GAGCAGGACTTGGCTTGCAATGGT-3’ as described (de Vere et al., 2015). The point mutation introduces a novel MseI restriction site in a 998bp or 587bp gDNA or cDNA region respectively. Homozygous point mutants were identified by PCR amplification of this 998bp region containing the novel restriction site followed by digestion with MseI restriction enzymes at 37 °C for 1 hr. Upon gel electrophoresis, WT gDNA results in 3 bands of 422, 333 and 243 bp while *vps45-3* gDNA results in 4 bands of 422, 243,213 and 120 bp. Restriction digestion of amplified cDNA results in two bands of 328bp and 259 bp for *vps45-3* cDNA while the WT cDNA lacks this site and thus results in a single 587 bp band.

Complementation of *vps45-3* mutants was performed by introducing a binary vector containing the *VPS45* coding sequence driven by the *VPS45* endogenous promoter described in (Zouhar et al., 2009). Plants were transformed using *Agrobacterium tumefaciens* by the floral dip method (Clough and Bent, 1998). Complemented lines were identified by resistance to hygromycin (30 mg L^−1^) and MseI restriction digestion as described above. Homozygous transformant lines were identified by appearance of all three bands in the restriction profile and resistance to hygromycin in subsequent progeny of the primary transformants.

EYFP- RabF2a (Preuss et al., 2004) constructs were generously provided by Dr. Erik Nielsen. All constructs were introduced into Arabidopsis by the floral dip method (Clough and Bent, 1998). Transgenic plants were selected on kanamycin and were imaged by confocal microscopy using a YFP filter at excitation and emission wavelengths of 488 nm and 528 nm respectively.

### 2.2 *In vitro* pollen germination and pollen tube length measurement

Flowers were collected from Arabidopsis plants 1 to 2 weeks after bolting and dehydrated at room temperature for at least 2 hrs. Pollen was germinated on an agar medium containing 18% sucrose (IBI Scientific, IB37160), 0.01% boric acid, 1 mM MgSO_4_, 1 mM CaCl_2_, 1 mM Ca(NO_3_)_2_, and 0.5% agar, pH 7 (Li et al., 1999) at room temperature for 12 hours. It was then examined under a Zeiss MacroZoom light microscope (Carl Zeiss Inc., Jena, Germany) and photographed with a 35 mm camera. Pollen tube lengths were measured as the distance from the pollen grain to the pollen tube tip, using segmented line and length measurements with the ImageJ software (Schneider et al., 2012). Average length and standard deviations for 100 pollen tubes were calculated for 3 independent biological replicates, n = 100.

### 2.3 Root hair phenotyping

Five-day-old seedlings were mounted on a slide and imaged using a Zeiss AxioImager microscope (Carl Zeiss Inc., Jena, Germany) with a 20X objective with bright field and differential interference contrast (DIC). Root hair length quantification was carried out by using segmented line and length measurements with the ImageJ software. Average length and standard deviations among at least 100 root hairs were calculated. Root hairs were imaged in the root elongation zone and neighboring cells in the early maturation zone, while excluding the older maturation zone cells.

### 2.4 FM4-64 staining and brefeldin A treatment

FM4-64 staining was modified from (Dettmer et al., 2006). To test bulk endocytosis, 4-day-old seedlings were transferred to MS liquid medium containing 4 μM FM4-64 (Invitrogen, T3166) for 2 min and subsequently washed twice for 30 s each time in 0.5× MS liquid medium before visualization. For analyzing arrival of FM4-64 at Brefeldin A (BFA) bodies, 4-day-old seedlings were transferred to 0.5× MS liquid medium containing 35 μM BFA (Sigma-Aldrich, B7651) for an hour followed by a 10 min treatment with 4 μM FM4-64 plus 35 μM BFA and two subsequent washes of 30 s each. The root tips were visualized using a Leica SP5 confocal laser scanning microscope (Leica Microsystems, Wetzlar, Germany) at the Iowa State University Roy J Carver High Resolution Microscopy Facility, using a 63× oil immersion objective lens and excitation and emission wavelengths of 558 and 734 nm. Images were acquired under identical conditions for both genotypes with equal exposure, scan frequency and line average settings. A total of 15 seedlings from at least three independent replicates were observed for each treatment and genotype.

### 2.5 Subcellular fractionation

0.8 g of WT and *vps45-3* 7-day-old seedlings were collected and ground in 1 mL of cold extraction buffer (0.3 M Sucrose, 0.1 M Tris-HCl, 1 mM EDTA, pH 7.5), with protease inhibitor cocktail (Roche). This was followed by centrifugation at 2,800*g* for 5 minutes at 4^o^C. A 100 μl portion of the supernatant was kept as the total protein fraction and the rest of the supernatant was transferred to a new tube followed by centrifugation at 13,000*g* for 30 minutes at 4^o^C. The pellet was resuspended in 100 μl of extraction buffer and represents the P13 fraction, and the supernatant was transferred to ultra-centrifuge tubes and centrifuged at 100,000*g* for 30 minutes at 4^o^C. The supernatant was transferred to a new tube and represents the SUP fraction, and the pellet was resuspended in 100 μl of extraction buffer and represents the P100 fraction.

Protein fractions were dissolved in SDS loading buffer and analyzed by immunoblotting using the indicated antibodies (Zouhar et al., 2009).

### 2.6 Immunoprecipitation

Immunoprecipitation was done as previously described (Bassham et al., 2000) using antibodies also previously described (Zouhar et al., 2009). 5 grams of 4 - to 6 - week-old Arabidopsis leaves were ground in 15 ml cold extraction buffer (0.3 M Sucrose, 0.1 M Tris-HCl, 1 mM EDTA, pH 7.5) with protease inhibitor cocktail (Roche, 11836153001). The crude extract was passed through Miracloth to remove debris, followed by centrifugation at 1000*g* for 5 min at 4°C. To dissolve membrane proteins, 0.5% Triton X-100 (v/v) was added to the supernatant, followed by rocking at 4°C for 2-3 hours. The protein extract was then transferred to ultra-centrifuge tubes followed by centrifugation at 100,000*g* to pellet the insoluble material. The supernatant was transferred to new 15 mL tubes and anti-SYP41 (1:200) antibodies were added to the samples followed by 2 hours rocking at 4°C. Protein A Sepharose CL-4B (Sigma-Aldrich, GE17-0780-01) was prepared according to the manufacturer’s protocol. The samples were further rocked with 50 μl suspended prepared protein A Sepharose overnight at 4°C. Beads were collected by centrifugation at 200*g* for 5 minutes at 4°C and washed 3 times with PBS buffer with 0.1% (v/v) Triton X-100. Proteins were eluted in SDS loading buffer (62.5 mM Tris-HCl (pH 6.8), 2% (w/v) sodium dodecyl sulfate, 25% (v/v) glycerol, and 0.01% bromophenol blue). Eluted proteins were analyzed by immunoblotting using the indicated antibodies.

### 2.7 FDA and MDY-64 and propidium iodide staining

Fluorescein diacetate (FDA) staining was performed as previously described (Saedler et al., 2009). Seedlings were submerged in a solution of 40 μg FDA in water for 5 min, and then mounted on a slide. Confocal microscopic images of root hairs were obtained using a Leica confocal microscope (Leica Microsystems, Wetzlar, Germany) using 63× oil immersion objective lens after excitation of the dye at 488 nm and emission was detected between 520 and 560 nm.

MDY-64 staining was performed as described (Scheuring et al., 2015). Seedlings were submerged in a solution of 0.25 μM MDY-64 (Invitrogen, Y7536) in 0.5X liquid MS medium for 5 min. The seedlings were then rinsed in 0.5X liquid MS medium and mounted on a slide. Confocal images of root hairs were obtained using a 63× oil immersion objective lens after excitation of the dye at 451 nm using an Ar/Kr laser, and emission was detected at 497 nm.

### 2.8 Visualization of YFP localization

Five-day-old seedlings were transferred to a slide and imaged using a Zeiss AxioImager microscope (Carl Zeiss Inc., Jena, Germany) with a 40X objective with differential interference contrast and confocal microscope using an EYFP-specific filter.

## 3 Results

### 3.1 *vps45-3* mutant has a severe dwarf phenotype

**Figure 1:**
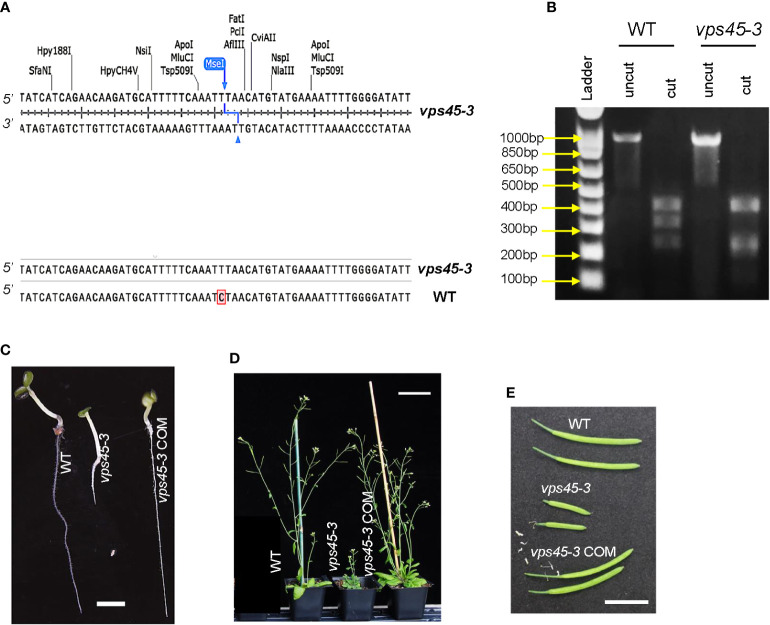
*vps45-3* mutants display severe phenotypic defects. **(A)** Coding sequence alignment of *VPS45* and *vps45-3* displaying the site of nucleotide substitution for *vps45-3. vps45-3* harbors a C-to-T substitution at the 851^st^ nucleotide of the *VPS45* coding sequence and this results in a novel MseI restriction site. **(B)** MseI restriction digest profiles of a 998 bp amplified gDNA fragment containing the mutation. *vps45-3* gDNA gives four bands of 422, 243, 213 and 120 bp upon MseI digest while the WT cDNA lacks this site and thus results in three bands of 422, 333, 243 bp upon digestion. **(C–E)** Phenotypes of WT, *vps45-3* and complemented (COM) *vps45-3*. **(C)** 7-day-old seedlings, grown on vertical plates on 0.5X MS medium, pH 6. Scale bar = 5 mm. **(D)** 30-day-old plants grown under long days showing the severe dwarf phenotype of *vps45-3* compared to the WT and *vps45-3* COM. Scale bar = 70 mm. **(E)** Siliques from 40-day-old long day-grown plants, Scale bar = 10 mm.

Arabidopsis VPS45 is essential for plant growth and development, as homozygous null mutants are inviable (Zouhar et al., 2009). As an alternative approach to determine the physiological roles of VPS45, a point mutation in VPS45 was recovered by a TILLING approach (Colbert et al., 2001) and designated as *vps45-3*. The mutation is a C-to-T substitution at the 851^st^ nucleotide of the *VPS45* coding sequence, leading to a serine-to-phenylalanine substitution at the 284^th^ amino acid position (**Figure 1A**). This mutation introduces a new MseI restriction site in the *vps45-3* coding sequence, allowing differentiation between mutant and wild-type alleles (**Figure 1B**).

*vps45-3* plants were dwarfed, with highly reduced sizes of many organs (**Figures 1C–E**, **S1**). similar to the previously reported *VPS45* RNAi lines (Zouhar et al., 2009), although less severe. To confirm that the observed phenotype results from mutation of *VPS45*, we introduced the *VPS45* cDNA driven by the native *VPS45* promoter into the *vps45-3* mutant to generate complementation lines (*vps45-3* COM) and assessed the plant phenotype. Growth and organ size defects of *vps45-3* plants were ameliorated by complementation with the *VPS45* transgene (**Figures 1C-E**, **S1**), confirming that the defects were caused by mutation of the *VPS45* gene and suggesting that the VPS45-3 protein has reduced function. The *vps45-3* plants were fertile and produced flowers and viable seeds without any noticeable abnormalities. This confirms that VPS45 is important for plant growth and that the *vps45-3* mutant is valuable to further analyze the function of VPS45.

### 3.2 VPS45 is important for cell expansion

**Figure 2:**
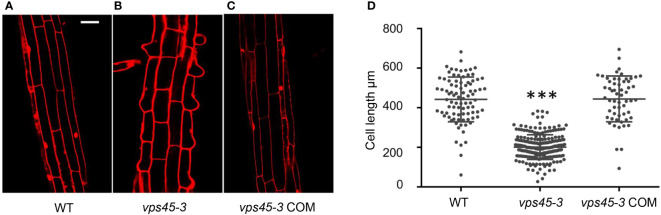
*vps45-3* exhibits reduced cell size. **(A–C)** WT, *vps45-3* and *vps45-3* COM roots were stained with propidium iodide (PI) and imaged by confocal microscopy. Scale bar =50 μm. **(D)** Plot of average cell size for WT, n = 88, *vps45-3*, n = 231, and *vps45-3* COM, n = 59. Cell length was computed using Image J software. Data are measurements of each individual cell, error bars show standard deviations, * shows statistically significant differences (P < 0.05) as determined by one-way ANOVA.

We reasoned that the dwarf phenotype of *vps45-3* plants could be due to reduced cell size, similar to RNAi plants. To analyze this possibility, 4-day-old seedling roots of WT, *vps45-3* and *vps45-3* COM were stained with propidium iodide (PI), which stains cell walls (Scheuring et al., 2015), and imaged by confocal microscopy. Cell size was significantly reduced in *vps45-3* compared to WT and *vps45-3* COM roots (**Figures 2A–D**). This was further visualized by using an agar imprinting method (Mathur and Koncz, 1997) to analyze the hypocotyl and root cells (**Figure S2**). Thus, a single ser-to-phe change in VPS45 causes cell expansion defects.

### 3.3 *vps45-3* mutants have reduced growth of root hairs and pollen tubes

**Figure 3:**
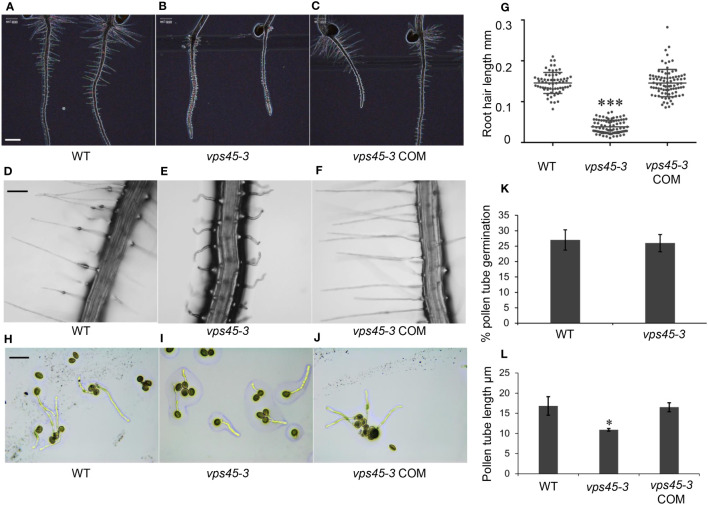
*vps45-3* has root hair and pollen tube defects. **(A–C)** Color inverted images of 5-day-old roots of WT, *vps45-3* and *vps45-3* COM to show root hair silhouettes. Scale bar = 500 μm. **(D–F)** Representative light microscopic images of 7-day old WT, *vps45-3* and *vps45-3* COM root hairs. Scale bar = 50 μm. **(G)** Root hair length of 7-day old WT, n= 66, *vps45-3*, n= 83 and *vps45-3* COM, n = 86 seedlings. Data are measurements of each individual root hair. Error bars show standard deviations, * shows statistically significant differences (P < 0.05), determined by one-way ANOVA. **(H–J)** Pollen grains from WT, *vps45-3* and *vps45-3* COM plants were germinated on medium overnight and examined under a light microscope. Scale bar = 50 μm **(K)** Percentage pollen germination comparison for WT and *vps45-3*. **(L)** Average pollen tube length comparison for WT, *vps45-3* and *vps45-3* COM. Data are measurements of average pollen tube length for three independent replicates. Error bars show standard deviations, * indicates statistically significant differences (P < 0.05), determined by one-way ANOVA.

We observed that *vps45-3* had a shorter main root compared to WT and had root hair defects (**Figures 3A-C**). Mutant root hairs were significantly shorter, wider and wavy compared with the root hairs of WT (**Figures 3D, E**). Complementation of the mutant rescued the root hair elongation defects (**Figures 3F, G**) confirming that the point mutation causes the observed root hair defects and that VPS45 is required for root hair cell expansion.

Root hairs and pollen tubes undergo tip growth, in contrast to other cells which undergo diffuse growth (Mathur and Hülskamp, 2001). Tip growth involves development of apical-basal polarity of the endomembrane system and rapid secretion at the tip of the developing root hairs and pollen tubes (Cole and Fowler, 2006; Rounds and Bezanilla, 2013; Šamaj et al., 2006). To determine if *vps45-3* has general defects in tip growth, we analyzed the growth of pollen tubes *in vitro*. Pollen from WT and *vps45-3* plants was plated onto pollen germination medium (Li et al., 1999) and incubated overnight to allow germination (**Figures 3H-J**). While no differences could be seen in the extent of germination between pollen from WT and mutant plants (**Figure 3K**), pollen tubes were significantly shorter in *vps45-3* compared to WT, and this defect was rescued in *vps45-3* COM lines (**Figure 3L**). These data suggest that VPS45 may play a role in tip growth in Arabidopsis.

### 3.4 The *vps45-3* mutation has no effect on interaction with and stability of the SYP41 SNARE complex

Our results indicate that *vps45-3* plants are dwarf with significant reduction in organ sizes, have defects in root hairs and pollen tubes, and show cell expansion defects. This suggests that the substitution of the serine to a phenylalanine, i.e. a polar to non-polar substitution, affects VPS45 function. Unlike a previously described VPS45 point mutant (*ben2*) (Tanaka et al., 2013), amino acid sequence alignment showed that the substituted amino acid in *vps45-3* is not conserved across different organisms (**Figure S3**), and that this amino acid may be important for VPS45 function in plants only.

**Figure 4:**
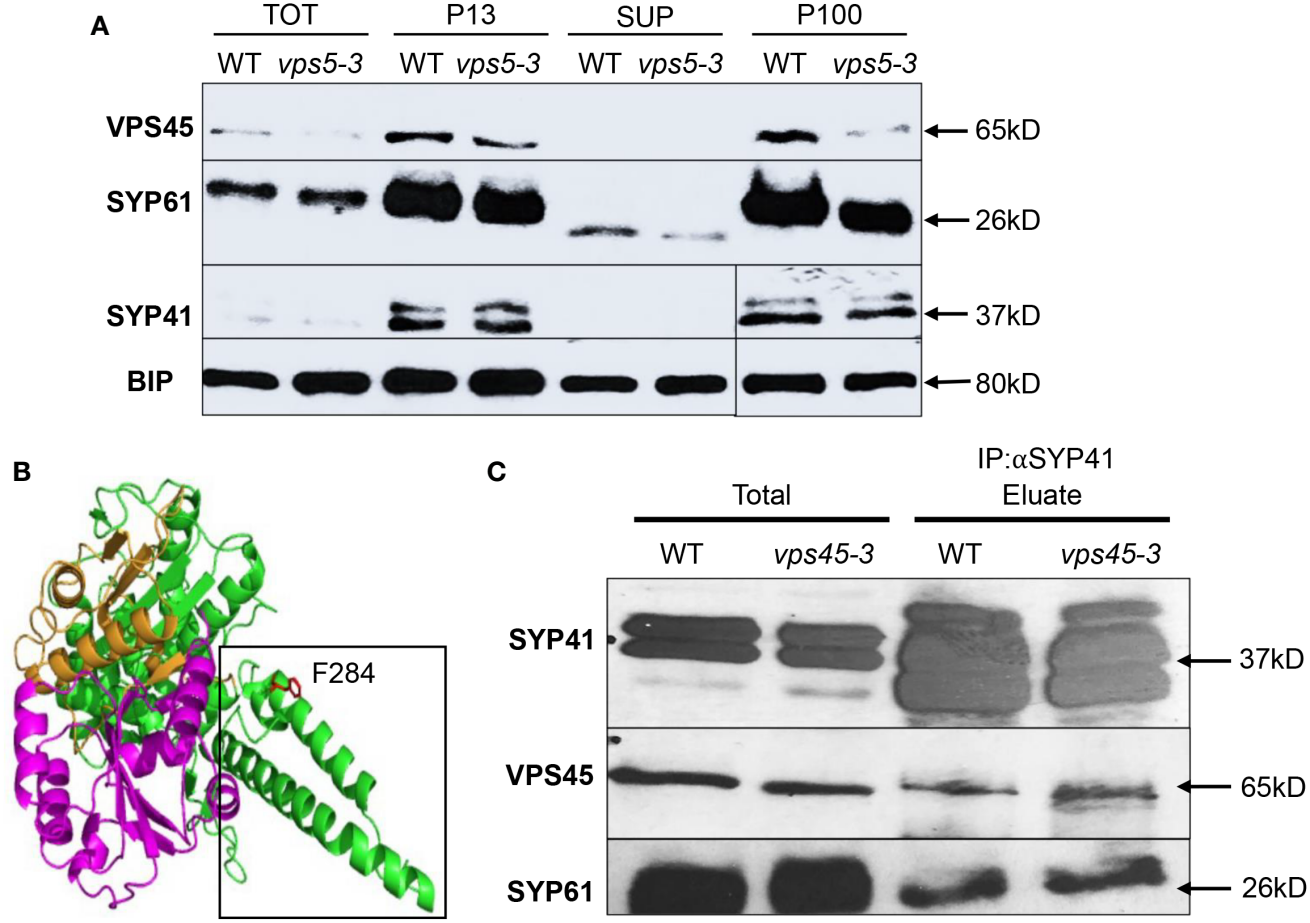
VPS45-3 maintains stability and interaction with SYP41 SNARE complex. **(A)** VPS45-3 maintains proper subcellular distribution and stability of SYP41 and SYP61. Total protein samples from 7-day-old wild-type and *vps45-3* plants were subjected to subcellular fractionation by centrifugation to obtain a total fraction (TOT) after centrifugation at 5,000*g*, low speed pellet (P13) after centrifugation at 13,000*g*, soluble fraction (SUP) and high-speed pellet (P100) after centrifugation at 100,000*g*. Aliquots of fractions were analyzed by SDS-PAGE followed by immunoblotting with the indicated antibodies. **(B)** Homology model of the predicted Arabidopsis VPS45-3 protein sequence modeled onto the crystal structure of c6MX1 from *Chaetomium thermophilum* (RCSB PDB). The mutated residue is shown in red and labeled and is in the 3a domain as shown by the black outline. **(C)** VPS45-3, SYP41, and SYP61 coimmunoprecipitate with SYP41 antibodies. Detergent solubilized membrane preparations from Arabidopsis leaves were subjected to immuno-isolation using SYP41 antibodies. Aliquots of total extracts and the eluate from the antibody column were analyzed by SDS-PAGE and immunoblotting with the indicated antibodies.

VPS45 has been implicated in regulating the stability and localization of its cognate SNARE complex (VTI12/SYP41/SYP61), as the levels of SYP41 were reduced in parallel to the levels of VPS45 in RNAi-silenced lines (Zouhar et al., 2009). To test the stability of both the SM protein and the SNARE complex we carried out subcellular fractionation of organelles from WT and *vps45-3* seedlings, followed by immunoblotting of different fractions with VPS45, SYP41 and SYP61 antibodies. The amount of SYP41, SYP61 and VPS45 was similar in WT and *vps45-3* in all fractions (**Figure 4A**), suggesting that both VPS45 and the SNARE proteins are stable in the mutant and that VPS45-3 can still associate with membranes.

To further understand the potential effect of the point mutation, we used homology modeling to fit the predicted Arabidopsis VPS45-3 protein sequence onto the crystal structure of c6MX1 from *Chaetomium thermophilum*, with a confidence score of 100.0 among all available protein structures. The mutation is in domain 3a of the protein (**Figure 4B**) and in close proximity to a region that is important for VPS45 interaction with other proteins (Eisemann et al., 2020).

Since binding of a SM protein to its cognate SNARE is required for SNARE complex function (Furgason et al., 2009; Shanks et al., 2012), we assessed whether defects in the mutant might be caused by altered interaction of VPS45-3 with SYP41 and SYP61. To test the interaction of VPS45-3 with SYP41 and SYP61, SYP41 was immunoprecipitated from WT or *vps45-3* plants using anti-SYP41 antibodies, and co-immunoprecipitation of VPS45 and SYP61 was assessed by immunoblotting (**Figure 4C**). The amount of VPS45 and SYP61 that co-precipitated with SYP41 was equivalent in WT and *vps45-3* mutant. This implies that the point mutation does not affect the interaction of VPS45-3 with SYP41 and SYP61 (**Figure 4C**).

### 3.5 Endocytosis and membrane arrival at the TGN are unaffected in the mutants

**Figure 5:**
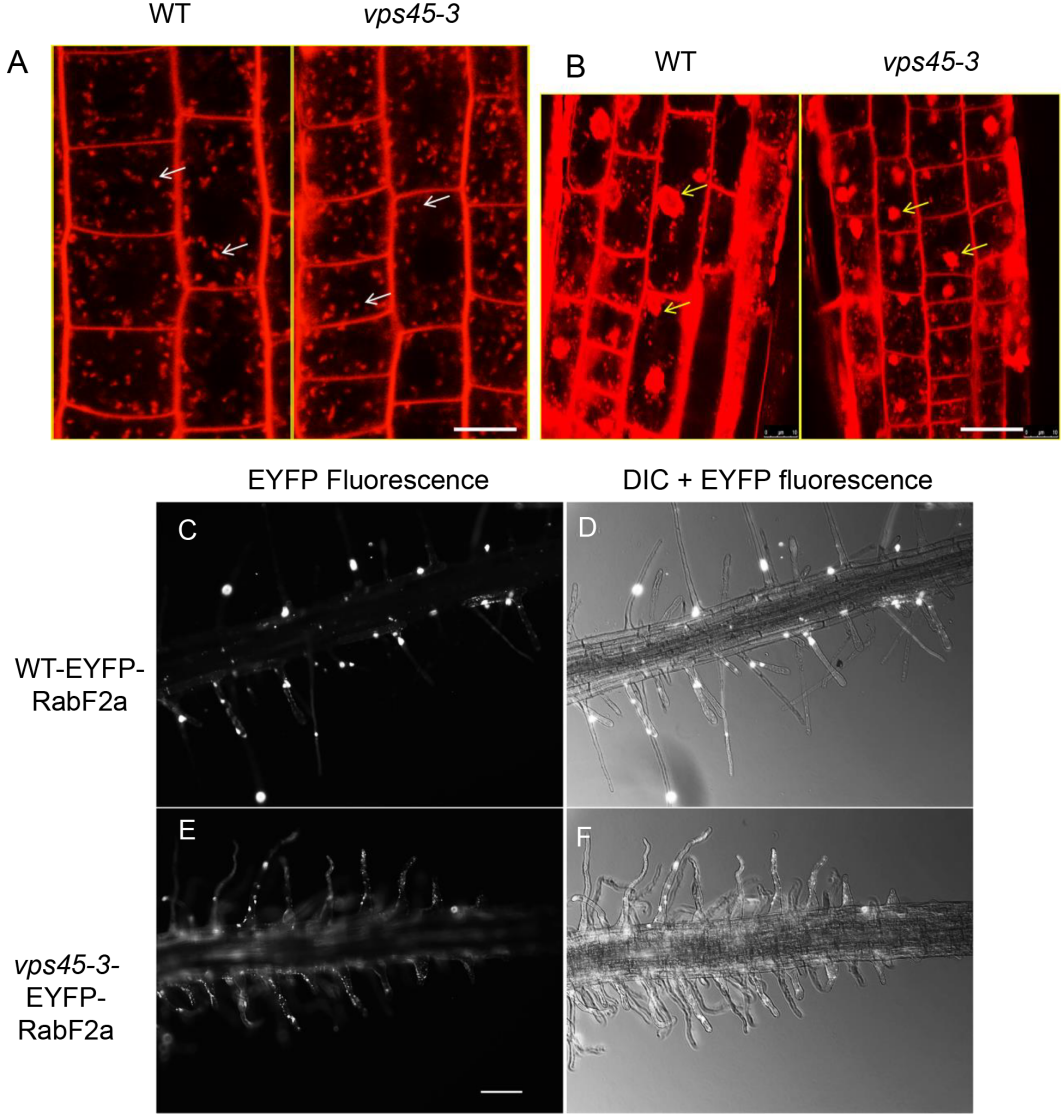
*vps45-3* mutants have unaltered bulk endocytosis and transport of membrane cargo from the plasma membrane to BFA bodies. **(A)** 4-day old seedling roots were treated with 4 μM FM4-64 in liquid 0.5x MS medium and imaged by confocal microscopy after 2 min of treatment. White arrows indicate early endosomes/TGN. **(B)** Root cells were treated with 35 μm BFA for 1 h, followed by a 10 min incubation with 4 μM FM4-64, showing arrival of FM4-64 at BFA bodies. Yellow arrows indicate BFA bodies. Scale bar = 10 μm. **(C–F)** Root hairs of 5-day-old seedlings expressing the early endocytic compartment marker EYFP-RabF2a were imaged using a Zeiss Upright microscope with 40X objective lens either with transmitted light or with epifluorescence illumination and appropriate EYFP filters. Scale bar = 500 μm.

Given that other *vps45* mutants have endocytic defects (Tanaka et al., 2013), we assessed whether endocytosis and recycling are affected in the *vps45-3* plants. This could explain the cell expansion phenotype owing to slower trafficking at the TGN during cell expansion (Gendre et al., 2015), particularly during root hair growth. We stained root cells with a FM4-64, a lipophilic styryl dye that is used as an endocytic tracer (Dettmer et al., 2006). The uptake of the FM4-64 was similar in both *vps45-3* and WT cells (**Figure 5A**), suggesting no major effect of the *vps45-3* mutation on internalization from the plasma membrane. We also tested whether membrane arrival at the TGN was affected in the mutants. We stimulated formation of TGN-endosomal aggregates by treating roots of 4-day old seedlings with Brefeldin A (BFA), a vesicle trafficking inhibitor, followed by incubation with FM4-64 and imaging with confocal microscopy. Labeling of the BFA compartments with the dye occurred at similar times in mutant and WT, suggesting that BFA body formation and arrival of membrane cargo at the TGN is not affected in the mutant (**Figure 5B**).

RabF2a is a Rab GTPase that localizes to early endocytic compartments in plants (Ueda et al., 2001; Preuss et al., 2004). We examined the distribution of RabF2a in root hairs of both WT and *vps45-3.* We transformed plants with an EYFP-RabF2a construct and observed three independently transformed lines by confocal microscopy. A similar distribution of EYFP-RabF2a was evident in both WT and *vps45-3* root hairs, with small punctate structures spread along the length of the root hair as previously reported (Preuss et al., 2004) (**Figures 5D–F**). The organization of the endosomal system therefore appears to be intact in *vps45-3*. Taken together, these data suggest that endocytosis and membrane trafficking from the PM to the TGN are unaffected in *vps45-3* mutants in both tip-growing and diffusely-growing cells.

### 3.6 *vps45-3* root hairs have vacuolar defects

The root hair and pollen tube phenotypes suggest that the *vps45-3* mutation might cause polarized tip growth defects. Polarized tip growth involves targeted deposition of cell wall and membrane material at the cell apex, and turgor pressure is a driving force for cell expansion *via* uptake of water into the vacuole (Cosgrove, 1993; Mendrinna and Persson, 2015). We reasoned that the root hair abnormality observed in *vps45-3* could be due to vacuole defects that disrupt tip growth. To test this, we analyzed root hair vacuoles by staining root hairs of five day old seedlings with the tonoplast marker MDY-64 (Scheuring et al., 2015), and imaged them using confocal microscopy.

**Figure 6:**
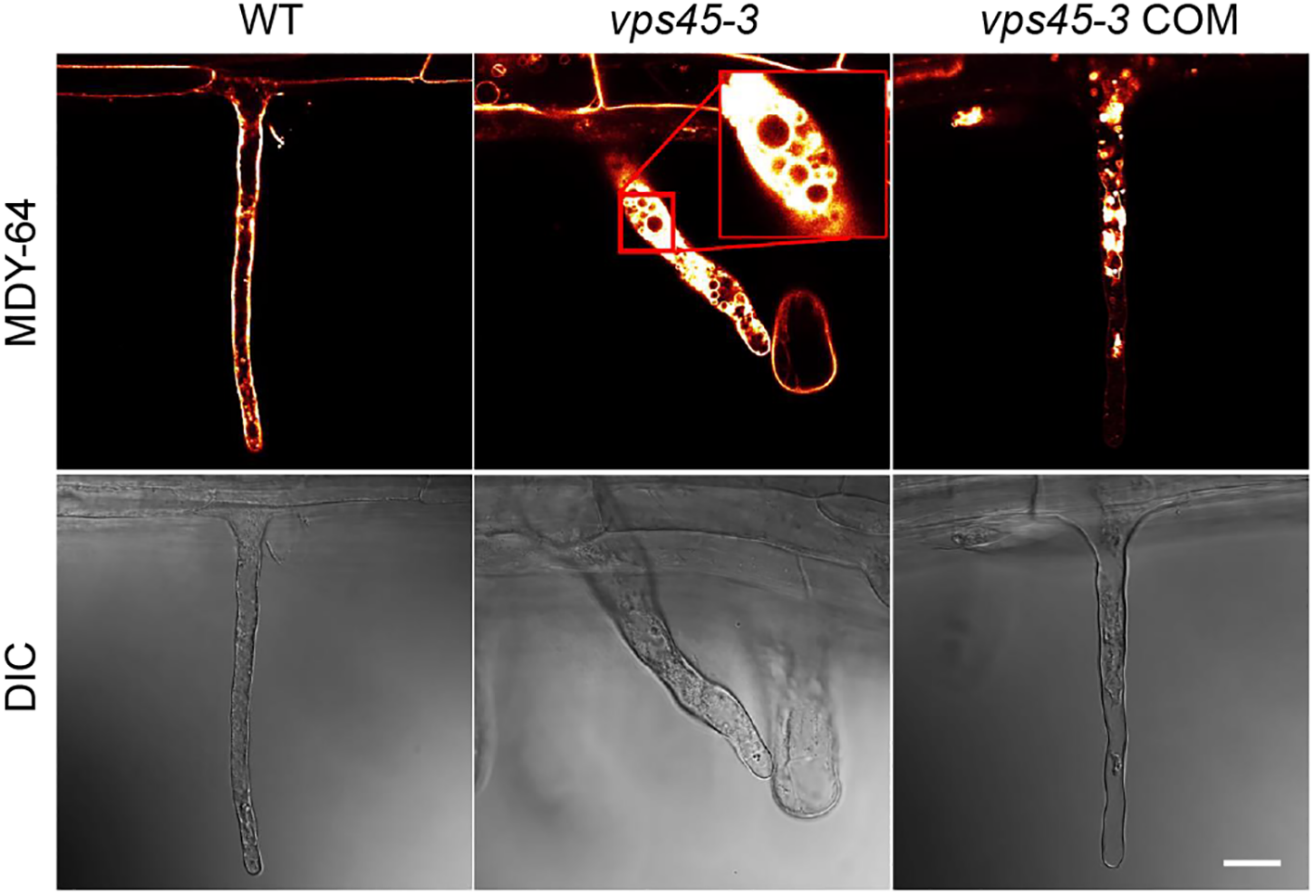
*vps45-3* seedling root hairs show vacuole defects. Root hairs from 5-day-old seedlings were treated with the tonoplast membrane stain MDY-64 and imaged using confocal microscopy. Scale bar = 20 μm, DIC indicates differential interference contrast microscopy.

In elongating WT and *vps45-3* COM root hairs, the vacuole was seen to occupy most of the cell, whereas in *vps45-3*, the root hair cell was filled with cytoplasm, with numerous small vacuoles visible (**Figure 6**; **Movies M1**, **M2**). Staining root hairs with fluorescein diacetate (FDA), which labels the cytoplasm, leaving the unstained vacuole visible, supported the idea that *vps45-3* roots hairs have defects in vacuole morphology, as they appeared to have increased cytoplasmic staining (**Figure S4**). The changes in vacuolar morphology in root hairs therefore correlate with the cell expansion defects observed in *vps45-3.*

## 4 Discussion

We identified a new Arabidopsis *vps45* mutant, *vps45-3*, which harbors a point mutation causing a serine-to-phenylalanine substitution at the 284^th^ position of the VPS45 polypeptide chain. Previous studies revealed that a homozygous null allele of *VPS45* is male gametophytic lethal, and RNAi lines with reduced VPS45 protein levels were used to study the function of VPS45 in plant growth (Zouhar et al., 2009). Similar to previously reported RNAi lines (Zouhar et al., 2009), *vps45-3* showed a dwarf phenotype, with reduced organ size and cell expansion defects, thus confirming that this point mutation impacts the normal function of VPS45.

A fundamental step in vesicle trafficking is the interaction between the three t-SNAREs present on the target membrane and the v-SNARE present on the transport vesicle membrane (Parlati et al., 2000). SM proteins function as chaperones to enable SNARE complex assembly, typically by binding to the t-SNARE, which adopts an open conformation to expose the presumptive R-SNARE binding site (Eisemann et al., 2020; Zhang and Hughson, 2021). One possibility is that a mutation could change the conserved structure and thus affect the binding of VPS45-3 to its interaction partners. However, we showed that interaction between the mutant VPS45-3 protein and t-SNAREs SYP41 and SYP61 was unaffected, and therefore this is not the cause of the phenotypic defects. In yeast, Vps45 also interacts with the v-SNARE Snc2 (Shanks et al., 2012). The VPS45 cognate v-SNARE in Arabidopsis may be YKT6 (Chen et al., 2005), although this has not been shown *in vivo*. Further work is required to investigate whether the point mutation affects the interaction with the v-SNARE and the formation of the *trans*-SNARE complex.

Although our results show no change in interaction between VPS45-3 and cognate SNARE binding partners (SYP41 and SYP61), it is possible that the mutation might affect the interaction with other proteins. For example, the human VPS45 SM protein was shown to bind to the Rab5 effector Rabenosyn-5 (Nielsen et al., 2000). Further studies will be needed to ascertain any other interacting partners of VPS45 and the impact of the point mutation on such interactions.

VPS45 has been implicated in endocytic uptake of membrane cargo from the plasma membrane (Tanaka et al., 2013). Based on this, we hypothesized that the *vps45-3* mutant may also have defects in endocytosis. Analysis of FM4-64 uptake in *vps45-3* mutants suggested that bulk endocytosis is unaltered and studies with the fungal toxin Brefeldin A (Lippincott-Schwartz et al., 1991) suggested that membrane cargo arrival at the TGN-endosomal aggregates is also unaffected. The *vps45-3* phenotypes seen are therefore likely to be a result of defects in biosynthetic trafficking to the vacuole.

We identified defects in polarized tip growth of both root hairs and pollen tubes in *vps45*-*3.* In root hairs, these tip growth defects correlated with vacuolar morphology defects, in which multiple small vacuoles were seen. VPS45 is important for localization of VSRs and for targeting of ctVSDs (Zouhar et al., 2009). Defects in VSR recycling and sorting of ctVSD-containing cargo may affect vacuolar morphology and subsequently cell expansion, and vacuole enlargement is critical during root hair expansion (Galway et al., 1997; Grierson and Schiefelbein, 2002). The SNARE VTI13 (belonging to the same family as VTI12) localizes to the vacuole and the TGN and is speculated to play roles in trafficking to the vacuole. Interestingly, a *vti13* mutant has mislocalization of SYP41 and defective root hair growth (Larson et al., 2014). This supports a connection between vesicle fusion machineries at the TGN and vacuole and root hair growth. A recent report also demonstrated that an SM protein involved in secretion is required for pollen tube growth, further implicating SM proteins in tip growth (Beuder et al., 2022).

In summary, we identified a *vps45-3* mutant which, unlike the previously described RNAi lines, maintained VPS45 protein levels and interaction with the t-SNARES SYP41 and SYP61 and their stability. Consistent with previous studies using knockdown lines (Zouhar et al., 2009), *vps45-3* had reduced cell and plant size. We demonstrate a role for VPS45 in tip growth of root hair and pollen tubes and show that *vps45-3* root hairs have fragmented vacuoles, compared to WT root hairs which have a single large vacuole occupying most of the root hair volume. We propose that reduced function of VPS45 leads to vacuole defects, which may affect vacuolar turgor pressure and consequently cause tip growth defects (Mendrinna and Persson, 2015).

## Data availability statement

The original contributions presented in the study are included in the article/**Supplementary Material**. Further inquiries can be directed to the corresponding author.

## Author contributions

YM, RR and DB designed the experiments. YM and RR conducted the majority of the experiments. GS and YV performed pollen assays. WA performed cell size and root hair measurement. YM, RR and DB wrote the manuscript. All authors contributed to the article and approved the submitted version.

## Supplementary material

Movie 1 WT root hairs stained with MDY-64 and imaged by confocal microscopy.

Movie 2
*vps45-3* root hairs stained with MDY-64 and imaged by confocal microscopy.
