# Supplementary material for: VPS45 is required for both diffuse and tip growth of Arabidopsis thaliana cells
Source: Front Plant Sci. 2023 Feb 27;14:1120307. doi: 10.3389/fpls.2023.1120307 (PMC10009167; doi:10.3389/fpls.2023.1120307)
Supplement: Supplementary file 1 [file DataSheet_1.pdf]

Supplementary Material

Supplementary Figures

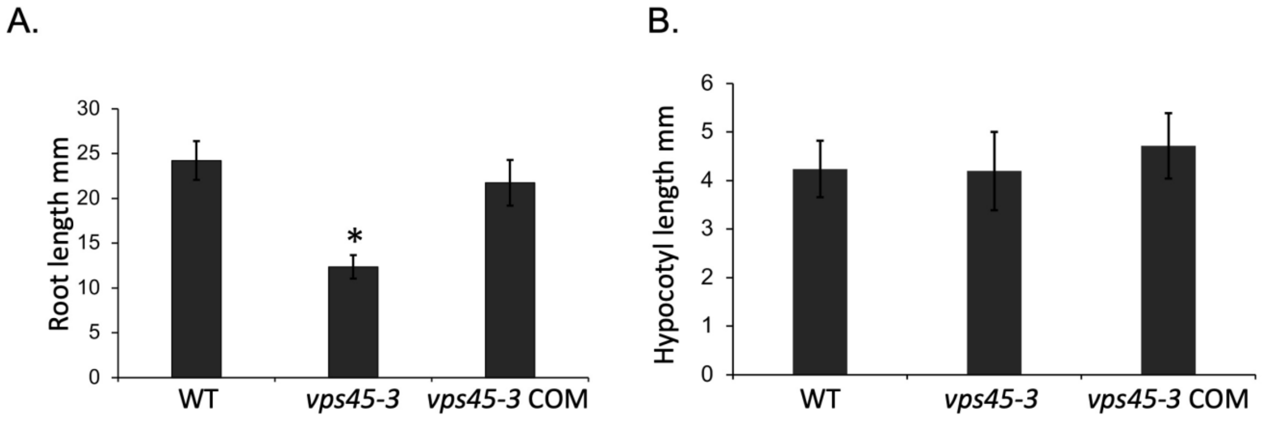

Supplementary Figure 1. *vps45-3* mutation affects root length. (A) and (B) Root and hypocotyl length respectively of seedlings of 7-day old WT, n= 4, *vps45-3*, n= 4 and *vps45-3* COM, n = 7, measured using ImageJ. Error bars show standard deviations, * shows statistically significant differences ($P < 0.05$), determined by one-way ANOVA.

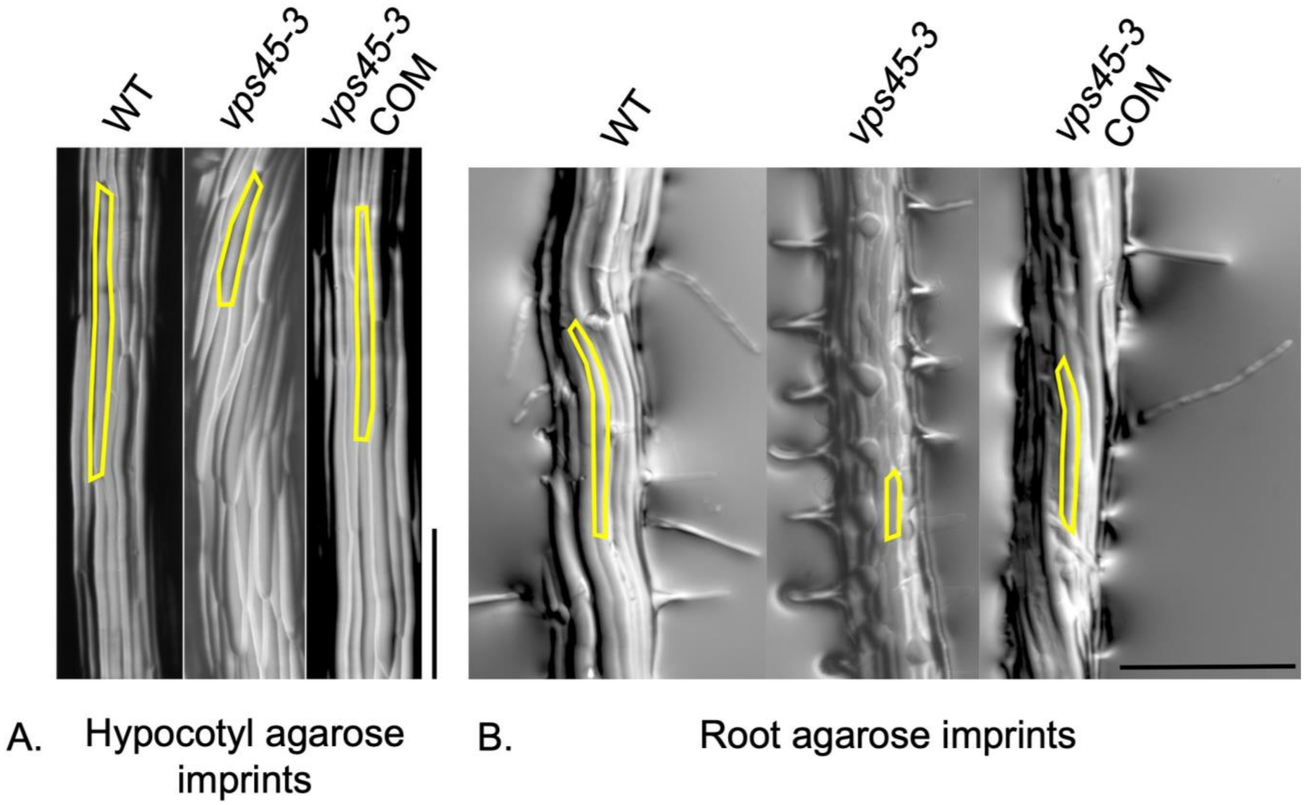

Supplementary Figure 2. *vps45-3* mutation leads to reduced cell size. Representative agarose imprints of hypocotyls (A) and roots (B) from 5-day-old dark grown seedlings of the indicated genotypes. Scale bar = 500 μm for hypocotyl and 200 μm for root imprints.

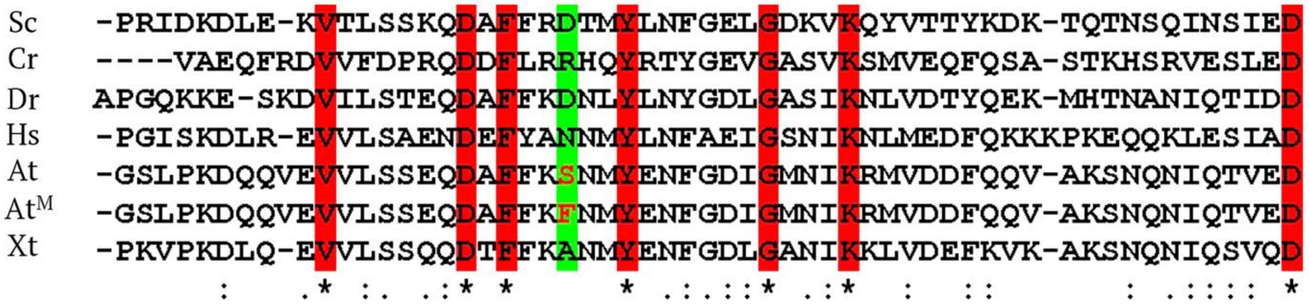

Supplementary Figure 3. Alignment of VPS45 protein sequences from various organisms around the *vps45-3* mutation site. Sc: *Saccharomyces cerevisiae*, Cr: *Chlamydomonas reinhardtii*, Dr: *Danio rerio*, Hs: *Homo sapiens*, At: *Arabidopsis thaliana*, At^M: *vps45-3* mutant sequence, Xt: *Xenopus tropicalis*.

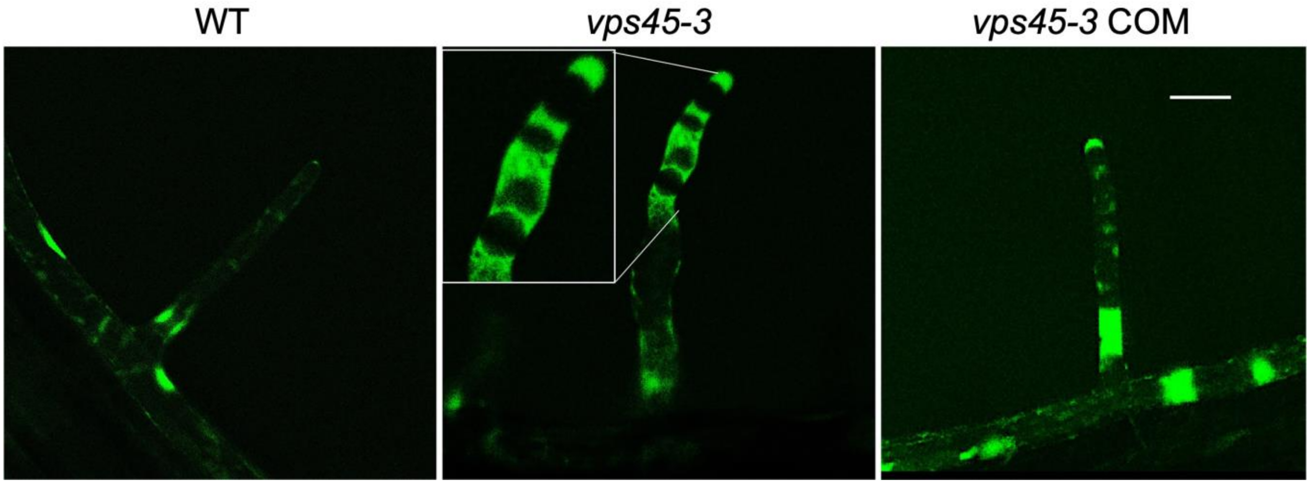

Supplementary Figure 4. *vps45-3* seedling root hairs have vacuole defects. Root hairs from 5-day old seedlings were treated with the cytoplasmic stain FDA and imaged using confocal microscopy. Fluorescent areas indicate cytoplasm; unlabeled areas correspond to the vacuole, from which the stain is excluded. Scale bar = 20 μm.
